# Motor imagery drives the effects of combined action observation and motor imagery on corticospinal excitability for coordinative lower-limb actions

**DOI:** 10.1038/s41598-024-63758-6

**Published:** 2024-06-06

**Authors:** Neza Grilc, Ashika Chembila Valappil, Neale A. Tillin, Omar S. Mian, David J. Wright, Paul S. Holmes, Federico Castelli, Adam M. Bruton

**Affiliations:** 1 Department of Life Sciences, Brunel University London, HNZW 271, Heinz Wolff Building, Uxbridge, UB8 3PH UK; 2 School of Life and Health Sciences, University of Roehampton, London, UK; 3 School of Psychology, Manchester Metropolitan University, Manchester, UK; 4 Department of Sport and Exercise Sciences, Manchester Metropolitan University, Manchester, UK

**Keywords:** Neuroscience, Cognitive neuroscience, Neurophysiology

## Abstract

Combined action observation and motor imagery (AOMI) facilitates corticospinal excitability (CSE) and may potentially induce plastic-like changes in the brain in a similar manner to physical practice. This study used transcranial magnetic stimulation (TMS) to explore changes in CSE for AOMI of coordinative lower-limb actions. Twenty-four healthy adults completed two baseline (BL_H_, BL_NH_) and three AOMI conditions, where they observed a knee extension while simultaneously imagining the same action (AOMI_CONG_), plantarflexion (AOMI_COOR-FUNC_), or dorsiflexion (AOMI_COOR-MOVE_). Motor evoked potential (MEP) amplitudes were recorded as a marker of CSE for all conditions from two knee extensor, one dorsi flexor, and two plantar flexor muscles following TMS to the right leg representation of the left primary motor cortex. A main effect for experimental condition was reported for all three muscle groups. MEP amplitudes were significantly greater in the AOMI_CONG_ condition compared to the BL_NH_ condition (*p* = .04) for the knee extensors, AOMI_COOR-FUNC_ condition compared to the BL_H_ condition (*p* = .03) for the plantar flexors, and AOMI_COOR-MOVE_ condition compared to the two baseline conditions for the dorsi flexors (*p*s ≤ .01). The study findings support the notion that changes in CSE are driven by the imagined actions during coordinative AOMI.

## Introduction

Action observation (AO) refers to observing human movement via video or live demonstration^1^, while motor imagery (MI) refers to the internal mental rehearsal of human movement execution^2^. There is a wealth of research demonstrating that interventions based on AO or MI can improve motor learning and execution in domains such as sport and neurorehabilitation^3–5^. According to motor simulation theory^6^, both AO and MI activate some shared neural pathways of the motor system as motor execution^7^. Therefore, this provides a potential Hebbian-based mechanism through which these motor simulation interventions improve movement execution and learning in the absence of overt physical practice of the movement. In support of this assertion, increased cortico-motor activity has been demonstrated for both AO and MI using a range of neuroscientific modalities (i.e., fMRI, EEG, TMS;e.g.,^7–11^. Collectively, this neuroscientific evidence indicates similar, but not identical, brain activation patterns for AO, MI and movement execution, providing mechanistic support for the efficacy of these techniques for motor learning and performance across populations and contexts^12^.

Over the last decade, research focus has shifted away from examination of independent AO and MI to instead explore the combined and simultaneous use of AO and MI (AOMI) as an intervention to improve motor skill performance and (re)learning (e.g.,^13–15^). In practice, *congruent* AOMI involves watching a movement demonstration whilst at the same time imagining the feeling of executing an identical movement in the same perspective^16,17^. Given that AO and MI recruit overlapping but distinct cortical pathways involved in motor execution^7^, the combination of these two motor simulation processes (i.e., *congruent* AOMI) is proposed to result in additive benefits in motor (re)learning via stronger and more widespread activity in brain regions associated with movement planning, production and control^17^. This proposition received partial support from a recent meta-analysis^18^ that found evidence for *congruent* AOMI facilitating corticospinal excitability (CSE) and improving movement outcomes compared to AO and control conditions, but not MI conditions. The authors^18^ concluded that, when repeated, *congruent* AOMI likely improved motor execution by inducing plastic-like changes in the motor system in a similar manner to physical practice. Whilst a growing body of literature provides empirical support for *congruent* AOMI*,* Vogt et al.^19^ outlined a spectrum of AOMI states where MI serves different roles during AO when they are executed simultaneously. This spans from *congruent* AOMI (i.e., identical content and visual perspective for AO and MI components of AOMI) to *conflicting* AOMI (i.e., opposing content and/or visual perspective for AO and MI components of AOMI). In between these AOMI states lies *coordinative* AOMI (i.e., *similar* content and/or visual perspective for AO and MI components of AOMI) whereby the observed or imagined actions can vary on a range of factors (e.g., action, modality, agency, speed, and perspective) that influence the relatedness of the two actions, from highly related to less related, during AOMI.

From a theoretical perspective, two contrasting hypotheses have been proposed to explain the underlying processes for these different AOMI states. First, Eaves and colleagues proposed the Dual-Action Simulation Hypothesis (DASH) account for AOMI^15,20^, which suggests that a person will generate separate motor representations for the observed and imagined actions and maintain these as two parallel sensorimotor streams when they engage in AOMI. For *congruent* AOMI, where the content and visual perspective are the same, these two motor representations are likely to merge as one sensorimotor stream, producing more widespread activity in the premotor cortex compared to AO or MI alone. For *conflicting* AOMI, visuo-motor representations of the observed and imagined movements are proposed to compete as separate sensorimotor streams, potentially producing similar cortico-motor activity as AO or MI alone depending on the relevance of the different simulated movements to the ongoing movement plan. For *coordinative* AOMI, visuo-motor representations of the observed and imagined movements may merge or compete, depending on the amount of transferable sensorimotor information between the two simulated movements, and the relevance of these to the ongoing movement plan.

Meers et al.^22^ proposed the Visual Guidance Hypothesis (VGH) as an alternative account for AOMI. The VGH argues that the imagined action is prioritized during AOMI, and that the observed action either serves as an external visual primer for the imagined action if displaying *the same movement,* or is ignored if displaying *another movement*. For fully *congruent* AOMI, the VGH proposes that the increased cortico-motor activity reported in previous literature (see^18^ for a recent meta-analysis) is likely due to the formation of a stronger motor representation for the imagined movement due to the priming effect of the observed movement. For both *conflicting* and *coordinative* AOMI, the VGH proposes that a motor representation will only be formed for the imagined movement due to prioritization of this component, meaning cortico-motor activity will be similar to that for independent MI. Both the DASH^15,20^ and VGH imply that *congruent* AOMI, if repeated, will lead to greater improvements in motor skill learning when compared to independent AO or MI by causing increased activation in motor regions of the brain over either in isolation. However, the two hypotheses differ in their predictions for *coordinative* AOMI. Specifically, the DASH predicts *coordinative* AOMI will lead to motor learning benefits for both the observed and imagined movements due to the formation of parallel sensorimotor representations for the two simulated movements, whereas the VGH proposes that benefits will only be attained for the imagined movement due to prioritization of imagery during *coordinative* AOMI.

Single-pulse TMS is the most prevalent neuroscientific modality adopted in the AOMI literature^18^. Only two studies, however, have empirically investigated *coordinative* AOMI to-date^20,22^. Both studies used TMS to examine the neurophysiological markers for *coordinative* AOMI using simple finger movements, and reported somewhat conflicting findings. Bruton et al.^20^ used a coordinative AOMI task in which participants observed index finger abduction–adduction movements whilst imagining little finger abduction–adduction movements. They reported that CSE was facilitated in the muscles that control both the observed index finger and imagined little finger during *coordinative* AOMI, when controlling for visual attention on the index finger. However, when Meers et al.^22^ used a similar AOMI task that alternated between AO or MI of little finger abduction–adduction and simultaneous MI or AO of index finger abduction–adduction, they found that CSE was only facilitated for the imagined finger movement, with no such facilitation for the observed finger movement during *coordinative* AOMI (termed incongruent AOMI in their paper). The findings from the two studies align with the specific predictions of the DASH and VGH, respectively, suggesting both hypotheses warrant further investigation of neurophysiological markers in the context of *coordinative* AOMI.

This study aimed to test the DASH^15,20^ and VGH^22^ propositions for AOMI by comparing neurophysiological markers of engaging in *congruent* AOMI and two types of *coordinative* AOMI. In this experiment, *congruent* AOMI (AOMI_CONG_) involved simultaneous observation and imagery of a knee extension movement in the same visual perspective. The two types of *coordinative* AOMI varied based on the movement parameter selected to relate the imagined movement to the observed knee extension movement. The first type of *coordinative* AOMI utilized plantarflexion of the foot as the imagined movement based on this being typically coupled with a knee extension movement when the body is propelled forward or upward by the legs, such as during the late stance phase in running and propulsion phase in jumping (i.e., AOMI_COOR-FUNC_). The second type of *coordinative* AOMI utilized dorsiflexion of the foot as the imagined movement because this causes distal portions of the foot to rotate in the same direction as the shank segment of the lower leg during knee extension, when both movements are viewed from the transverse plane (i.e., AOMI_COOR-MOVE_). If the propositions of the DASH^15,20^ hold true, CSE facilitation will be greatest in the simultaneously observed and imagined knee extensor (KE) muscle group for the *congruent* AOMI condition, but will also be facilitated in both the observed KE muscle group and imagined plantar flexor (PF) or dorsi flexor (DF) muscle groups for the two types of *coordinative* AOMI compared to both baseline conditions. If the predictions of the VGH^22^ are correct, CSE facilitation will be greatest in the simultaneously observed and imagined KE muscle group for the *congruent* AOMI condition, but will only be facilitated in the imagined PF or DF muscle groups for the two types of *coordinative* AOMI compared to both baseline conditions.

## Results

### MEP amplitude data

#### Knee extensor (KE) muscle group

**Figure 1:**
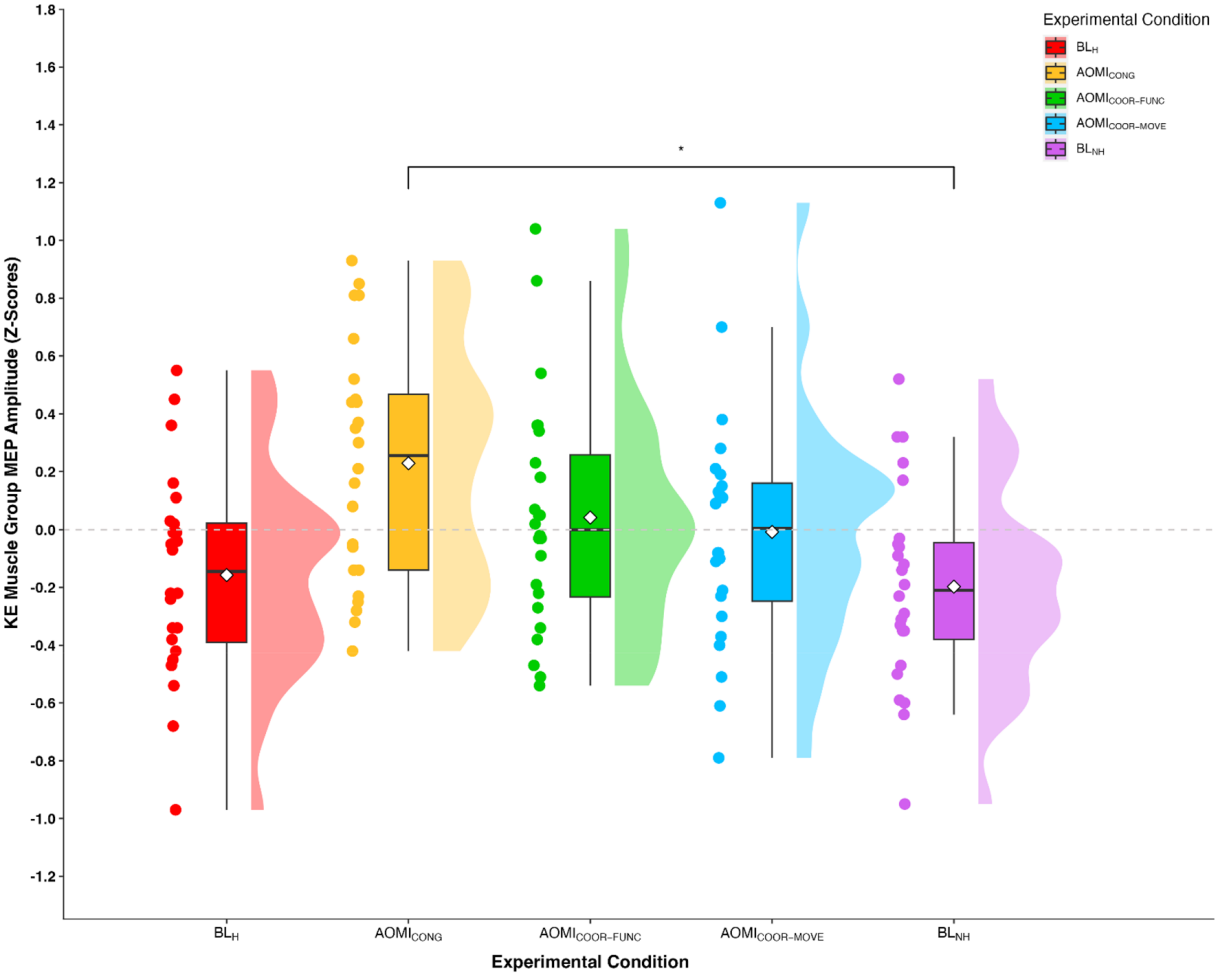
Box and violin plot with raw data points displaying z-score normalized MEP amplitudes from the knee extensor muscle group for the five experimental conditions. *Note:* Thick horizontal black lines represent the median average and white diamonds represent the mean average for each box plot. Individual participant data points are represented by circular markers. BL_H_—human baseline; BL_NH_—non-human baseline; AOMI_CONG_—congruent action observation and motor imagery; AOMI_COOR-FUNC_—functionally coordinative action observation and motor imagery; AOMI_COOR-MOVE_—movement coordinative action observation and motor imagery; *p < .01.

In the KE muscle group, peak-to-peak values for the baseline EMG data showed no main effect of experimental condition, F_(2.98,68.55)_ = 1.05, *p* = 0.38, $\eta$_p_^2^ = 0.04. Z-score MEP amplitude data violated the assumptions of sphericity, χ^2^_(9)_ = 21.06, *p* = 0.01, and thus a Greenhouse–Geisser correction was applied. The one-way repeated measures ANOVA on the KE group *z*-score MEP amplitude data reported a significant large main effect of experimental condition, F_(3.18,73.13)_ = 3.77, *p* = 0.01, $\eta$_p_^2^ = 0.14. Pairwise comparisons (see Table 1 for hypotheses-focused pairwise comparisons) indicated that z-score MEP amplitudes were significantly larger in the AOMI_CONG_ condition compared to the BL_NH_ condition (*p* = 0.04) and approached a significantly larger value compared to the BL_H_ condition (*p* = 0.056). No other significant differences were reported for pairwise comparisons (Fig. 1).

**Table 1:** Mean, standard error (SE), confidence interval (CI), and alpha values (***p***) for focal post-hoc pairwise comparisons between z-score normalized MEP amplitudes from the KE, PF and DF muscle groups for the five experimental conditions.

Condition	Muscle	Mean	SE	95% CI	vs	Condition	Mean	SE	95% CI	*p*
AOMI_CONG_	KE	0.23	0.08	[0.06, 0.40]	vs	BL_H_	−0.16	0.07	[−0.31, −0.01]	.056
					vs	BL_NH_	−0.20	0.07	[−0.34, −0.05]	.**04**
AOMI_COOR-FUNC_	KE	0.04	0.08	[−0.13, 0.21]	vs	BL_H_	−0.16	0.07	[−0.31, −0.01]	.99
					vs	BL_NH_	−0.20	0.07	[−0.34, −0.05]	.71
	PF	0.35	0.12	[0.10, 0.61]	vs	BL_H_	−0.18	0.06	[−0.31, −0.05]	**.03**
					vs	BL_NH_	−0.16	0.07	[−0.30, −0.01]	.07
AOMI_COOR-MOVE_	KE	−0.01	0.08	[−0.18, 0.17]	vs	BL_H_	−0.16	0.07	[−0.31, −0.01]	.99
					vs	BL_NH_	−0.20	0.07	[−0.34, −0.05]	.99
	DF	0.36	0.10	[0.14, 0.57]	vs	BL_H_	−0.16	0.05	[−0.26, −0.06]	**.003**
					vs	BL_NH_	−0.17	0.07	[−0.31, −0.02]	**.008**

BL_H_—human baseline; BL_NH_—non-human baseline; AOMI_CONG_—congruent action observation and motor imagery; AOMI_COOR-FUNC_—functionally coordinative action observation and motor imagery; AOMI_COOR-MOVE_—movement direction coordinative action observation and motor imagery.

Significant values are in bold.

#### Plantar flexor (PF) muscle group

**Figure 2:**
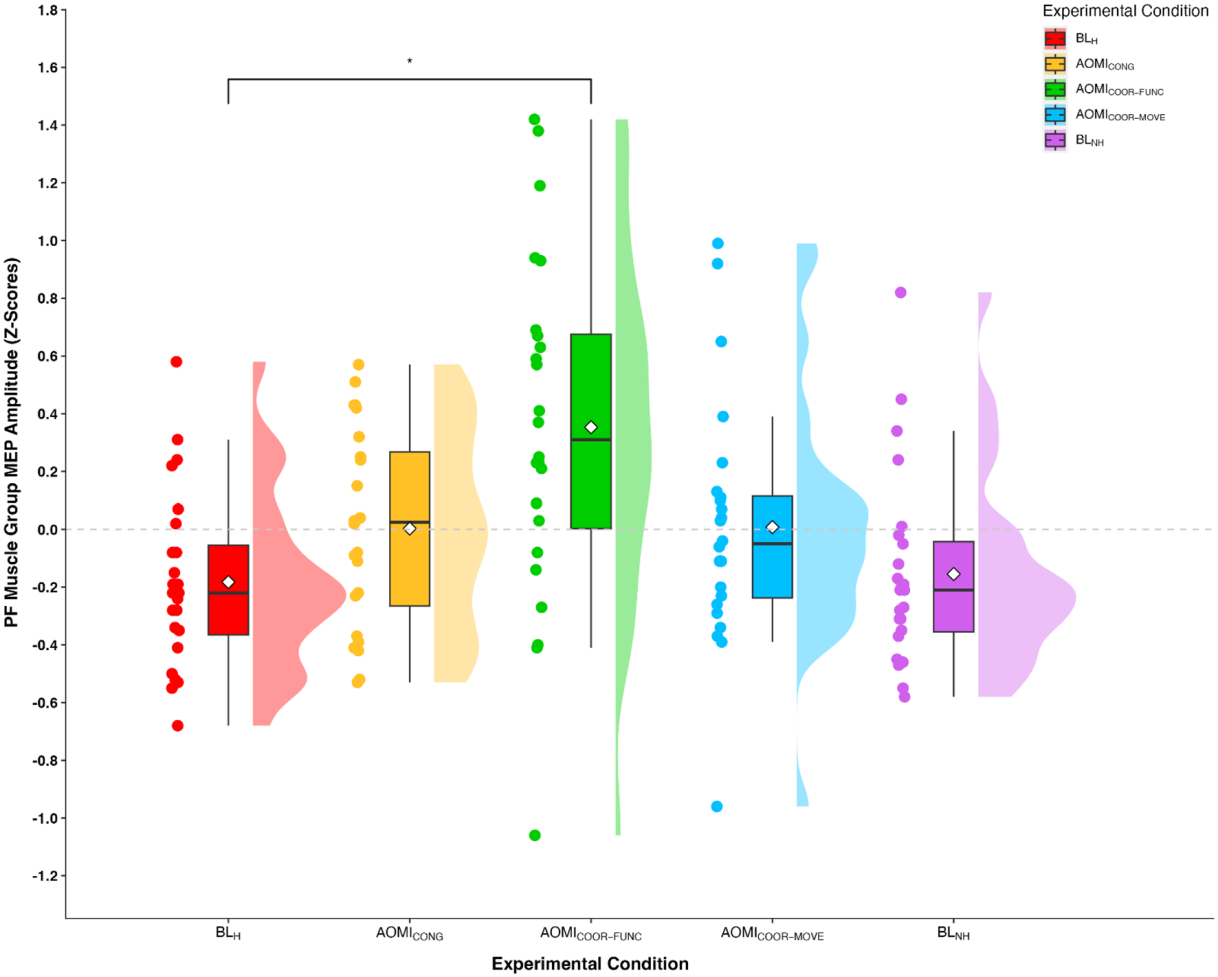
Box and violin plot with raw data points displaying z-score normalized MEP amplitudes from the plantar flexor muscle group for the five experimental conditions. *Note:* Thick horizontal black lines represent the median average and white diamonds represent the mean average for each box plot. Individual participant data points are represented by circular markers. BL_H_—human baseline; BL_NH_—non-human baseline; AOMI_CONG_—congruent action observation and motor imagery; AOMI_COOR-FUNC_—functionally coordinative action observation and motor imagery; AOMI_COOR-MOVE_—movement direction coordinative action observation and motor imagery; *p < .05.

In the PF muscle group, peak-to-peak values for the baseline EMG data approached a significant main effect of experimental condition, F_(2.49,57.23)_ = 2.57, *p* = 0.07, $\eta$_p_^2^ = 0.10, but pairwise comparisons revealed no significant differences between experimental conditions. Z-score MEP amplitude data violated the assumptions of sphericity, χ^2^_(9)_ = 32.11, *p* < 0.001, and thus a Greenhouse–Geisser correction was applied. The one-way repeated measures ANOVA on the PF group *z*-score MEP amplitude data demonstrated a significant large main effect of experimental condition, F_(2.66,61.11)_ = 5.12, *p* = 0.004, $\eta$_p_^2^ = 0.18. Pairwise comparisons (see Table 1 for hypotheses-focused pairwise comparisons) showed that z-score MEP amplitudes were significantly larger in the AOMI_COOR-FUNC_ condition compared to the BL_H_ condition (*p* = 0.03) and approached a significantly larger value compared to the BL_NH_ condition (*p* = 0.07). No other significant differences were reported for pairwise comparisons (Fig. 2).

#### Dorsi flexor (DF) muscle group

**Figure 3:**
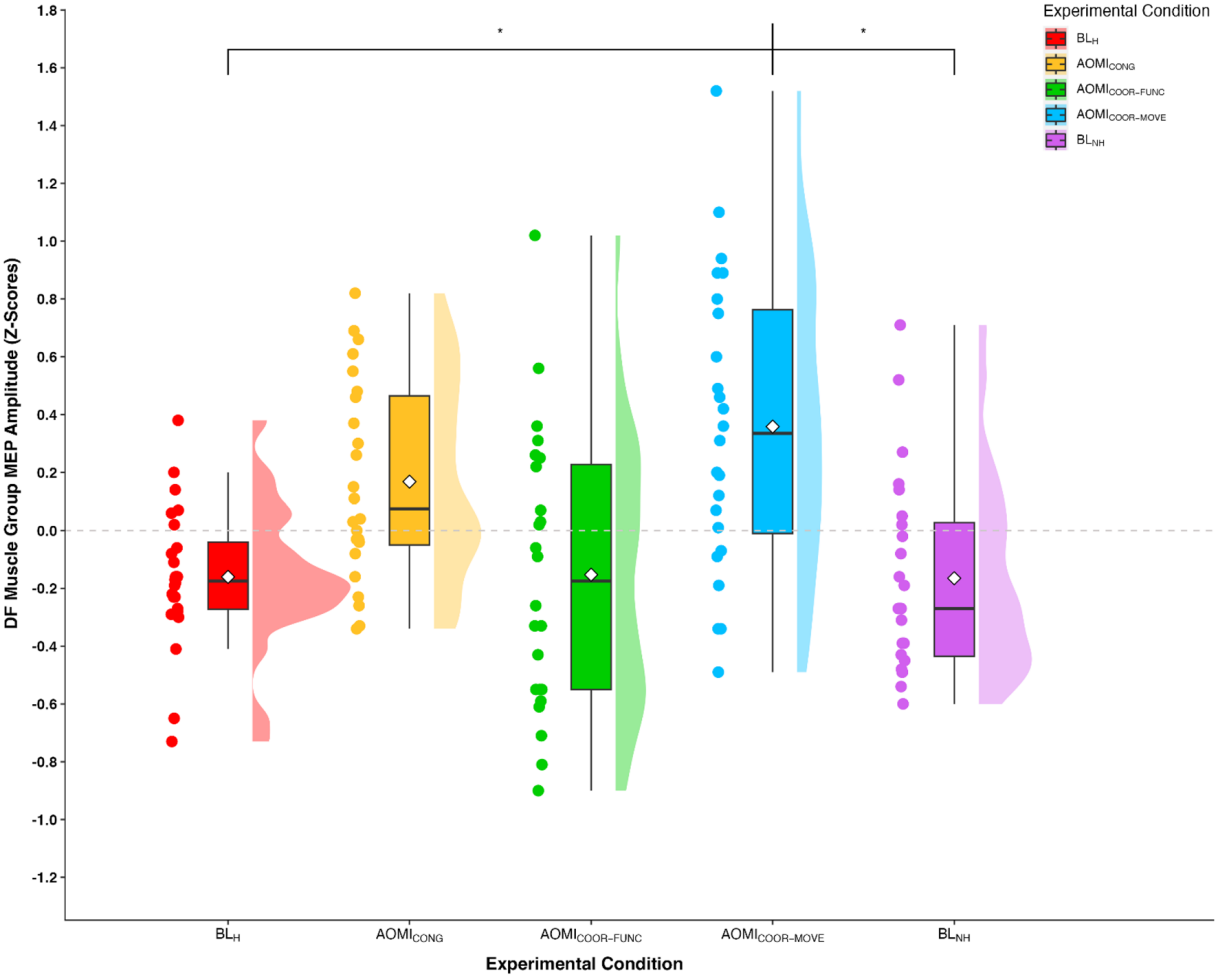
Box and violin plot with raw data points displaying z-score normalized MEP amplitudes from the dorsi flexor muscle group for the five experimental conditions. *Note:* Thick horizontal black lines represent the median average and white diamonds represent the mean average for each box plot. Individual participant data points are represented by circular markers. BL_H_—human baseline; BL_NH_—non-human baseline; AOMI_CONG_—congruent action observation and motor imagery; AOMI_COOR-FUNC_—functionally coordinative action observation and motor imagery; AOMI_COOR-MOVE_—movement direction coordinative action observation and motor imagery; *p < .01.

In the DF muscle group, peak-to-peak values for the baseline EMG data showed no main effect of experimental condition, F_(2.43,55.97)_ = 0.62, *p* = 0.57, $\eta$_p_^2^ = 0.03. Z-score MEP amplitude data violated the assumptions of sphericity, χ^2^_(9)_ = 27.75, *p* < 0.001, and thus a Greenhouse–Geisser correction was applied. The one-way repeated measures ANOVA on the *z*-score MEP amplitude data demonstrated a significant large main effect of experimental condition, F_(4,92)_ = 7.16, *p* < 0.001, $\eta$_p_^2^ = 0.24. Pairwise comparisons (see Table 1 for hypotheses-focused pairwise comparisons) showed that z-score MEP amplitudes were significantly larger in the AOMI_COOR-MOVE_ condition compared to BL_H_ (*p* < 0.01) and BL_NH_ (*p* < 0.01), and approached a significantly larger value compared to the AOMI_COOR-FUNC_ condition (*p* = 0.07). No other significant differences were reported for pairwise comparisons (Fig. 3).

### Social validation data

The Greenhouse–Geisser corrected one-way repeated measures ANOVA results for self-reported perceived ease/difficulty of generating and maintaining kinesthetic imagery, F_(1.932, 44.434)_ = 2.60, *p* = 0.09, $\eta$_p_^2^ = 0.102, showed no differences between AOMI experimental conditions (i.e., AOMI_CONG_, AOMI_COOR-FUNC_, AOMI_COOR-MOVE_). This was also reflected in the social validation interview data gathered from participants that reflected mixed perceptions about kinesthetic imagery ability across these conditions. Specifically, seventeen participants (70.83%) suggested kinesthetic imagery was easiest or joint-easiest in the AOMI_CONG_ condition (e.g., “I think the knee extension as this was already on display, yeah, so I mean when you were doing the other two then I struggled a little bit” [participant 4]). Nine participants (37.50%) suggested kinesthetic imagery was easiest or joint-easiest in the AOMI_COOR-FUNC_ condition (e.g., “Plantar flexion. I think it was just being able to time it and just the thought of it, I think it’s quite a regular movement for me, so yeah that one seemed probably the best” [participant 7]). Nine participants (37.50%) suggested kinesthetic imagery was easiest or joint-easiest in the AOMI_COOR-MOVE_ condition (e.g., “Yeah, I found it easier to focus on like, pulling my toes up specifically for some reason, and like my ankle” [participant 12]). Three participants (12.50%) felt there was no difference in kinesthetic imagery ease between AOMI conditions (e.g., “In hindsight, I don’t think I found any of them particularly hard… Practicing the movement before each experimental block made imagery pretty easy for all of the conditions” [participant 14]).

All participants felt like they were looking at a same-sex leg, with fourteen participants (58.33%) feeling like this was their own leg (e.g., “It did feel like the video was my leg, it felt like I was seeing my own leg” [participant 22]), and seven participants (29.17%) feeling like they were watching someone else’s leg (e.g., “And then thinking about whose leg is this? “it’s not my leg” so yeah maybe a little bit difficult but generally there is a feeling but you're wondering whether it's the right feeling” [participant 24]). Twenty-one participants (87.5%) used first person perspective MI, suggesting that the presentation format for the AO stimuli made this feel natural (e.g., “I was just picturing my legs moving looking down” [participant 15]), and facilitated their kinesthetic imagery (e.g., “I know that that's not my leg on the screen, it’s very logical this is not me, but I could imagine what those movements would feel like in my leg with those muscle groups” [participant 5]).

No participants reported engaging in any kind of imagery in the two baseline conditions, indicating that they only looked at the stimulus, as instructed (e.g., “I was just very focused on keeping my eyes on the fixation cross and not losing focus” [participant 4]). When combining BL_H_ and BL_NH_ as an initial (block 1) and final (block 5) baseline, there was no difference in z-score MEP amplitude data based on timing in the session for KE (*t*(23) = − 1.63, *p* = 0.12, *d* = −0.33), PF (*t*(23) = −0.50, *p* = 0.62, *d* = −0.10) or DF muscle groups (*t*(23) = −0.67, *p* = 0.51, *d* = −0.14). This supports the rigor of the experimental design, as baseline levels returned despite engagement in three blocks of AOMI tasks between the two baseline blocks (e.g., “Obviously we've been [doing] loads of imagery stuff and I think that requires more thinking so at the last one I was a bit happy that I was just looking at the fixation cross, not imagining anything in particular while looking at the fixation cross” [participant 4]).

## Discussion

**Figure 4:**
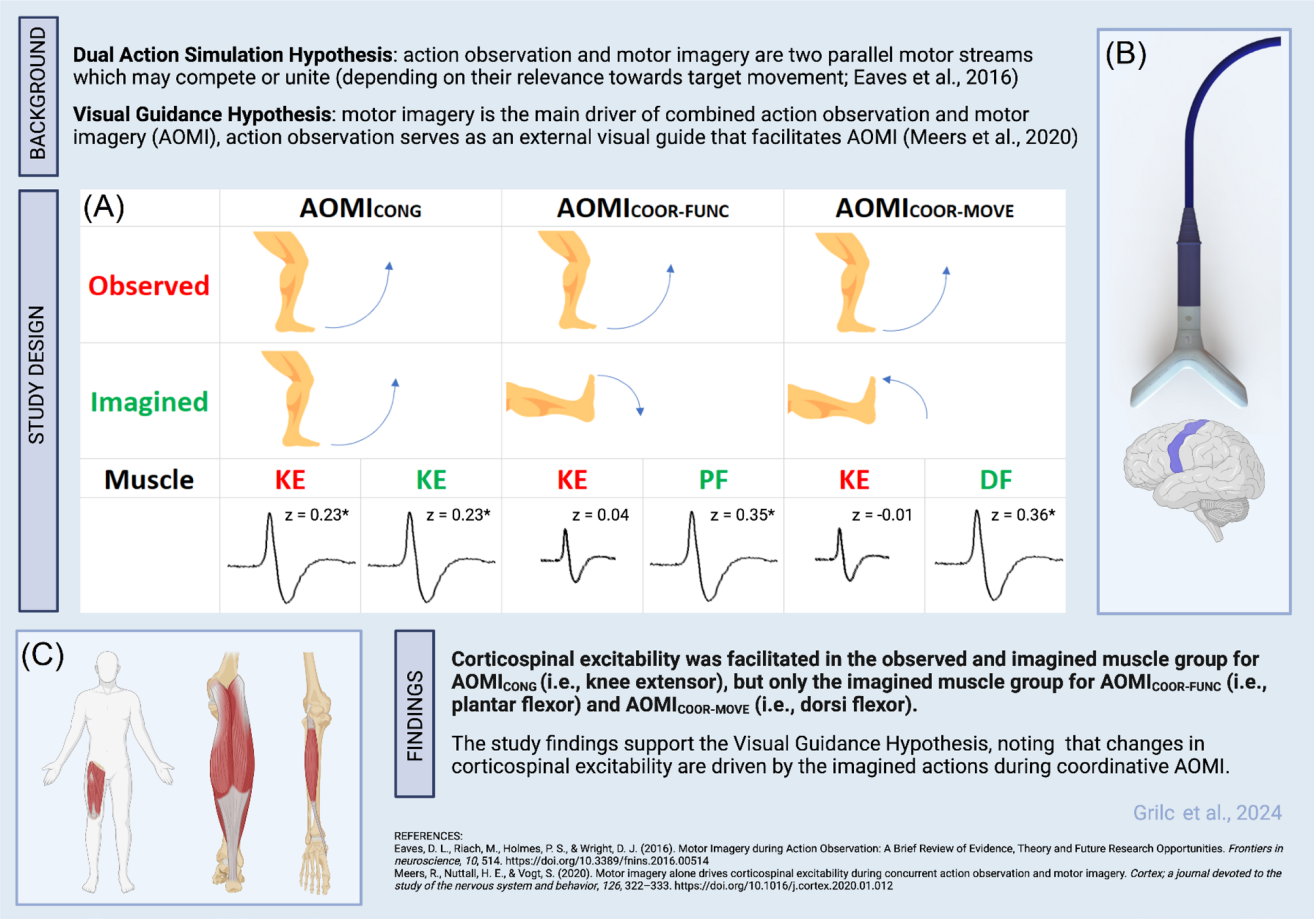
A graphical abstract depicting the main components of the study protocol and findings related to the tested Dual-Action Simulation Hypothesis and Visual Guidance Hypothesis. Twenty-four healthy adults completed two baseline and three AOMI experimental conditions. (**A**) During the AOMI experimental conditions, participants observed a knee extension movement while simultaneously imagining a knee extension (AOMI_CONG_), a plantarflexion (AOMI_COOR-FUNC_), or a dorsiflexion (AOMI_COOR-MOVE_) movement. (**B**) Single-pulse transcranial magnetic stimulation was delivered to the right leg representation of the left motor cortex at 110% resting motor threshold whilst the participants engaged in the baseline and AOMI experimental condition trials. (**C**) Electromyographical activity was recorded for all trials from two knee extensor, one dorsi flexor and two plantar flexor muscles, allowing for the assessment of motor evoked potentials related to the observed and imagined muscles during AOMI experimental conditions. * denotes a significant difference between the z-score transformed MEP amplitude in the AOMI experimental condition and at least one of the baseline conditions.

The aim of this study was to test the DASH^15,20^ and VGH^22^ propositions for AOMI by comparing neurophysiological markers of engaging in *congruent* AOMI and two types of *coordinative* AOMI. Social validation data were also collected to further explain the cognitive processes underpinning any differences in neurophysiological response during these different types of AOMI. Overall, the findings support the VGH as CSE was facilitated in the observed and imagined muscle group for AOMI_CONG_, but only the imagined muscle group for AOMI_COOR-FUNC_ and AOMI_COOR-MOVE_, compared to baseline conditions (Fig. 4).

In this experiment, *congruent* AOMI involved the simultaneous observation and imagery of an identical KE movement with MI in the same visual perspective to the AO perceptual cues. Our findings for *congruent* AOMI are consistent with the propositions of both the DASH^15,20^ and VGH^22^, as MEP amplitudes were significantly larger in the KE muscle group during AOMI_CONG_ compared to the BL_H_ condition and approached significance compared to the BL_NH_ condition. This finding is in agreement with a body of literature showing CSE facilitation for *congruent* AOMI compared to control conditions (e.g.,^18,20,28^). The DASH proposes that this CSE facilitation is due to the merger of the two motor representations for AO and MI of the KE movement as one sensorimotor stream, producing widespread activity in the premotor cortex. This is likely due to *congruent* AOMI increasing activity in a greater number of shared brain areas for AO and MI, as well as increasing activity in areas solely recruited during AO and MI for the KE movement (e.g.,^7,21^). The VGH proposes that this CSE facilitation for *congruent* AOMI occurs because a stronger motor representation is formed for the imagined movement due to the priming effect of the observed movement. The social validation data lends support to this assertion, as participants typically found it easiest to generate and maintain imagery during the AOMI_CONG_ condition employed in this experiment. The inclusion of a pure MI condition and imagery ability characteristic checks within each block would allow further conclusions to be made. However, single-pulse TMS is not the ideal neuroscientific method for determining brain activation patterns related to the observed and imagined movements during *congruent* AOMI. Whole-brain neuroscientific modalities with increased spatial resolution should be combined with pattern-classification analysis methods (e.g., fMRI multi-voxel pattern analysis) to distinguish between contributions for these different motor simulation processes during *congruent* AOMI^20^.

The findings reported here for *congruent* AOMI have important implications for motor (re)learning across settings such as neurorehabilitation and sport. If repeated as part of a training program, the increased pre-motor and motor activity reported for *congruent* AOMI is likely to promote Hebbian modulation of intracortical and subcortical excitatory mechanisms through similar synaptic plasticity mechanisms to those observed following physical practice^29^. Consequently, researchers have advocated the use of fully *congruent* AOMI interventions to improve motor function and learning (e.g.,^17,18,30^). Current behavioral evidence supports the efficacy of using *congruent* AOMI to improve movement outcomes across different contexts, populations and motor skills^18^. Movement outcome benefits have been demonstrated for fine motor skills such as dart throwing^13^ and gross motor skills such as whole-body balance tasks^31^ in both neurotypical (e.g.,^32,33^) and neurodivergent populations (e.g.,^30,34,35^). Longitudinal research incorporating both neurophysiological and behavioral measures is now required to verify the extent to which repeated engagement in *congruent* AOMI promotes functional connectivity and plasticity changes within the brain, and the association between these neural adaptations and any motor performance and learning improvements after a *congruent* AOMI intervention period.

Two *coordinative* AOMI conditions were adopted in this experiment, where the participants observed a KE movement and simultaneously imagined the feelings and sensations of either a PF (AOMI_COOR-FUNC_ condition) or DF movement (AOMI_COOR-MOVE_ condition). Our findings for *coordinative* AOMI provide support for the propositions of the VGH^22^ and oppose the propositions of the DASH^15,20^. MEP amplitudes were significantly larger in the imagined PF muscle group during the AOMI_COOR-FUNC_ condition compared to the BL_H_ condition, and imagined DF muscle group during the AOMI_COOR-MOVE_ condition compared to both BL_H_ and BL_NH_ conditions. However, there was no difference in MEP amplitudes for the observed KE muscle group in the AOMI_COOR-FUNC_ or AOMI_COOR-MOVE_ conditions compared to both BL_H_ and BL_NH._ This finding aligns with the results of Meers et al.^22^ that showed CSE was only facilitated for imagined muscles during *coordinative* AOMI of finger movements, and disagrees with the findings of Bruton et al.^20^ that showed CSE facilitation for both observed and imagined muscles when controlling for visual attention during *coordinative* AOMI of finger movements. Based on the propositions of the VGH, this finding reflects the prioritization of imagery during AOMI, meaning the motor representation is maintained for the imagined movement (i.e., PF or DF) and disregarded for the observed movement (i.e., KE) during *coordinative* AOMI in this study. This study provides the most robust neurophysiological evidence to-date supporting the VGH explanation over the DASH^15,20^ for coordinative AOMI effects. Specifically, it appears that parallel sensorimotor streams cannot be maintained for observed and imagined actions during *coordinative* AOMI; instead the imagined action is prioritized and the action observation component serves as a visual guide to facilitate motor imagery processes.

Studies report CSE facilitation for *congruent* AOMI compared to pure MI (e.g.,^36^), suggesting that the observed movement acts as a primer for the generation and maintenance of more vivid imagined movements during AOMI of the same action. It is possible that AO performs the same facilitatory role during *coordinative* AOMI, as sensory and/or kinematic movement information is shared between the different observed and imagined actions. This is supported by the social validation questionnaire data from this study, as perceived kinesthetic imagery ability was rated as ‘somewhat easy/easy to feel’ and did not statistically differ across the three AOMI conditions. Indeed, participants suggested the observed movement acted as a trigger, and helped with timing of their imagery for the two *coordinative* AOMI conditions. It is not possible to make this conclusion objectively from the current study as a pure MI condition was not included for comparison. Therefore, future single-pulse TMS studies should compare the neurophysiological markers for *coordinative* AOMI against pure MI to see if an additive facilitation is reported for CSE.

The findings reported for *coordinative* AOMI have implications for motor (re)learning. Whilst *congruent* AOMI is recommended as the optimal action simulation intervention for (re)learning a specific movement^17^, *coordinative* AOMI has the capacity to benefit the learning of new actions and (re)learning of joint actions. The two types of *coordinative* AOMI employed in this study (i.e., AOMI_COOR-FUNC_ and AOMI_COOR-MOVE_) facilitated CSE in the imagined muscle. These *coordinative* AOMI conditions may provide a viable complementary training method to physical therapy in rehabilitation settings and may promote the (re)learning of actions that are currently impaired or missing from a person’s motor repertoire. For example, a post-stroke patient may benefit from observing videos of themselves accurately performing leg movements such as balance or jumping tasks with their non-affected limb, whilst simultaneously imagining the feelings and sensations associated with performing the same or similar leg movements with their impaired limb (e.g.,^37^). In such cases, *coordinative* AOMI could support motor (re)learning by promoting Hebbian plasticity in a similar manner to that described above for *congruent* AOMI. Alternatively, a youth footballer could observe a skilled age-grade footballer passing a football (i.e., similar to the KE movement used in this study), and imagine him/herself performing the same pass, as well as variations of the pass that require technical adaptations (i.e., including varied DF or PF movements, as used in this study) to learn new skills in co-active and interactive sporting settings. Given this is one of only three studies to investigate *coordinative* AOMI as an isolated AOMI state (see^20,22^ for two previous studies), research exploring the efficacy of *coordinative* AOMI as a motor skill performance and learning intervention is warranted to substantiate propositions that *coordinative* AOMI may be better than *congruent* AOMI for (re)learning complex serial movements, or in sports where interpersonal coordination is required^1,38^.

This study is the first to investigate the neurophysiological and perceived cognitive mechanisms associated with different types of *coordinative* AOMI. Although the findings of the current study align with the propositions of the VGH^22^, it is important to note that participants’ eye gaze was constrained by the use of a fixation cross during both experiments. The fixation cross used in this study was placed in a position that maximized the informational uptake from the observed movement^39^, whereas Meers et al.^22^ placed the fixation cross at a point not involved in either the observed or imagined finger movements for their study. Visual attentional strategies are reported to differ across AOMI states when visual attention is not constrained^20^, and this is likely motivated by the perceptual-cognitive demands of the task whereby visual attention is directed towards a location to serve a specific purpose. For example, a person may want to visually attend more to the observed movement to improve movement information uptake, or visually attend to the region involved with the imagined movement to help them generate the sensations involved with the movement more effectively during *coordinative* AOMI^20^. The varied CSE responses and implemented visual constraints adopted in this study and the two previous single-pulse TMS studies exploring *coordinative* AOMI^20,22^ raise this as a potential confound when making comparisons between, and drawing conclusions from, these studies. This is especially pertinent as visual attention serves as a key mechanism underlying the effects of both AO and MI^40^. Therefore, future studies should investigate the effect of different visual strategies on the neurophysiological markers associated with *coordinative* AOMI.

In conclusion, the findings of this study support the assertions of the VGH^22^ and oppose the suggestions of the DASH^15,20^ propositions for *coordinative* AOMI. Specifically, CSE was facilitated in the simultaneously observed and imagined KE muscle group for *congruent* AOMI, but only facilitated in the imagined PF and DF muscle groups for *coordinative* AOMI based on function (AOMI_COOR-FUNC_) and movement direction (AOMI_COOR-MOVE_), respectively. This study provides neurophysiological support for the use of *coordinative* AOMI as a novel alternative method for (re)learning of movements that extend beyond a learner’s repertoire, or for joint actions that require coordination with the movements of others.

## Methods

### Participants

Based on previous AOMI studies employing TMS (e.g.,^20,23,41^, twenty-four healthy adults aged 19–42 years (50% male, M_age_ = 26.21 ± 5.45 years, 91.67% right-handed) with normal or corrected-to-normal vision took part in this study (see Table 2 for detailed breakdown of study demographic information). Prior to the experiment, all participants provided their written informed consent and completed a screening pack including the TMS Adult Safety Screen (TASS;^42^), and the Vividness of Movement Imagery Questionnaire 2 (VMIQ-2;^43^). The factorial, concurrent and construct validity of the VMIQ-2 has been supported as a psychometric scale for motor imagery ability assessment^43^. The final study sample reported clear and reasonably vivid external (27.71 ± 9.67), internal (23.50 ± 9.75) and kinesthetic (24.79 ± 7.60) imagery ability (see Table 1). Due to the potential impact of menstrual cycle on cognitive processing of motor actions and subsequent motor cortex excitability^44^, female participants were tested during the follicular phase of their menstrual cycle (i.e., days 1–13 of a 28-day cycle).

**Table 2:** Demographic information of sample recruited to take part in this study.

	N	Age in years (Mean ± SD)	VMIQ-2 score (Mean ± SD)			TMS Method Details		
			Internal	External	Kinesthetic	OSP Location: Modal distance from Cz	Mean RMT Intensity	Mean EXP Intensity
Total	24	26.21 ± 5.45	23.50 ± 9.75	27.71 ± 9.67	24.79 ± 7.60	1 cm lateral (n = 8), 1 cm anterior (n = 9)	61 ± 11%	68 ± 12%
Male	12	28.50 ± 5.70	22.83 ± 9.84	31.50 ± 9.46	25.00 ± 7.30	0.5 cm lateral (n = 5), 1 cm anterior (n = 6)	57 ± 8%	63 ± 9%
Female	12	23.92 ± 4.27	24.17 ± 10.04	23.92 ± 8.64	24.58 ± 8.22	1.5 cm lateral (n = 6), 0.5 cm anterior (n = 7)	65 ± 13%	72 ± 14%

N = 24; OSP—optimal scalp position; RMT—resting motor threshold; EXP—experimental.

### Experimental design

**Figure 5:**
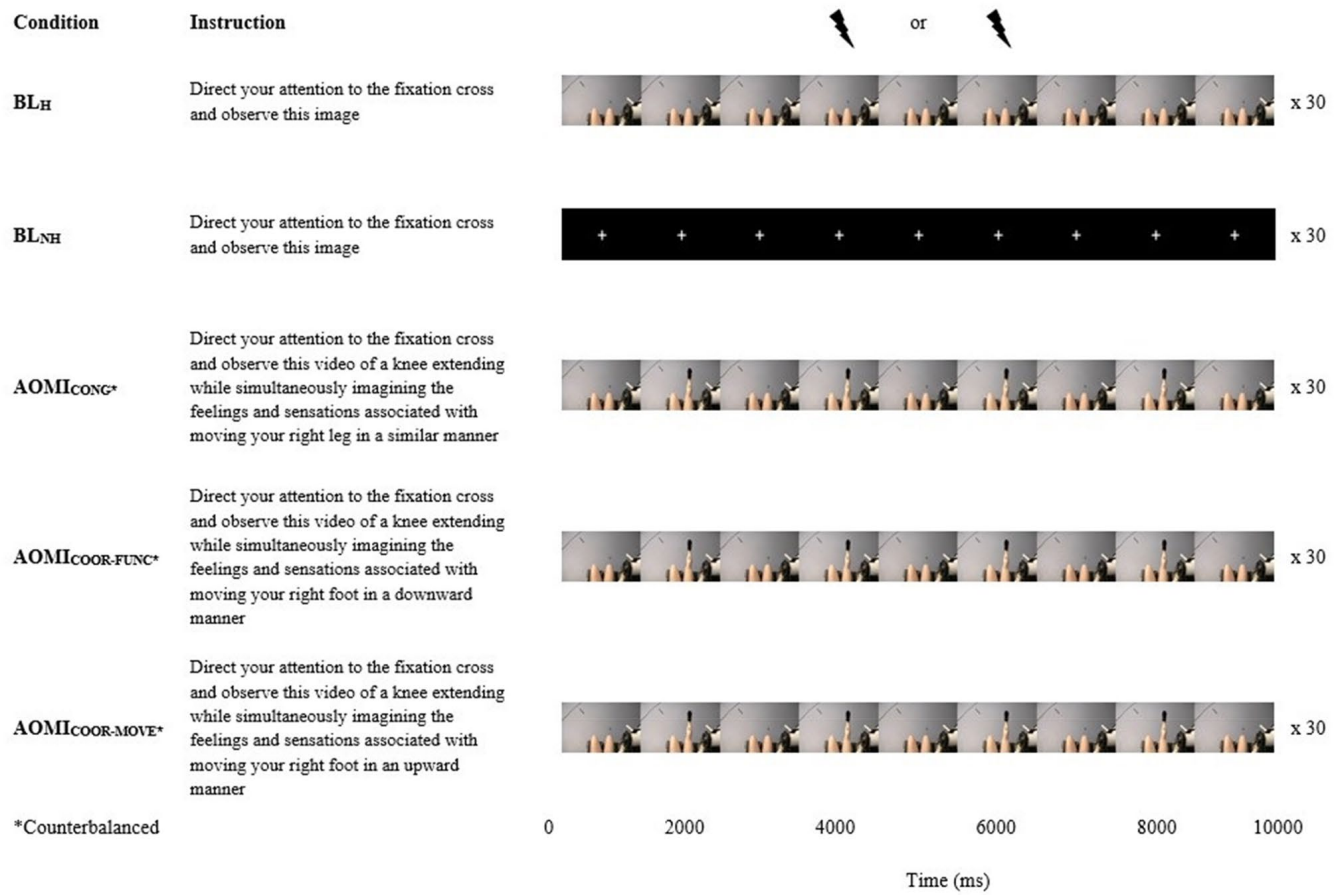
A Visual Representation of the Five Experimental Conditions. *Note:* The TMS was delivered at the point of maximum knee extension for either the second (4000ms) or third (6000ms) cycle of every AOMI trial, and at the same time point for trials in both baseline conditions. The ordering of the TMS delivery was randomized and counterbalanced across trials for each experimental block.

The study was conducted in accordance with ethical guidelines and the study approval was granted from the University of Roehampton Ethical Committee (LSC 21: 346). All procedures were reported using parts A, B and C of the Guidelines for Reporting Action Simulation Studies checklist^45^. A repeated measure design was employed where all participants completed five experimental conditions (Fig. 5): (i) a human baseline (BL_H_) condition where seated participants were instructed to observe a first person perspective (1PP) static scene of the legs presented with the knee flexed in a relaxed position (30 trials); (ii) a non-human baseline condition (BL_NH_) where participants were instructed to observe a white fixation cross presented against a black screen (30 trials); (iii) a *congruent* AOMI (AOMI_CONG_) condition where participants were asked to observe a 1PP video of a right-leg KE movement, while simultaneously imagining, from the same 1PP, the kinesthetic feelings and sensations involved with the same KE movement of their own right leg (30 trials); (iv) a *coordinative* AOMI condition based on the coupling of lower-limb movements during the performance of typical functional tasks such as walking and kicking (AOMI_COOR-FUNC_), where participants were asked to observe a 1PP video of a right-leg KE movement, while simultaneously imagining, from the same 1PP, the kinesthetic feelings and sensations involved with a PF movement of their own right foot (30 trials); and (v) a *coordinative* AOMI condition based on the direction of movement (AOMI_COOR-MOVE_) where participants were asked to observe a 1PP video of a right-leg KE movement, while simultaneously imagining, from the same 1PP, the kinesthetic feelings and sensations involved with a DF movement of their own right foot (30 trials). All participants completed 15 trials of the BL_H_ condition and 15 trials of the BL_NH_ as the first and last experimental blocks (experimental block 1 and 5). The AOMI conditions were completed in the middle experimental blocks (experimental blocks 2, 3, and 4) and the order of these was randomized and counterbalanced across the study sample.

### Experimental procedure

#### Surface electromyography (EMG) preparation and recording

Prior to EMG placement, the skin was prepared by shaving, cleaning (70% ethanol), and lightly abrading the area where EMG electrodes were placed. A single, bipolar silver-silver-chloride gel-electrode configuration (2-cm diameter, 2-cm inter-electrode distance; Dual Electrode, Noraxon, Arizona, U.S.A.) was placed over each of the following muscles of the right leg: vastus lateralis and vastus medialis (KE muscle group); soleus and gastrocnemius (PF muscle group); and tibialis anterior (DF muscle group). Electrode locations were determined using SENIAM 8 guidelines^46^. Noraxon wireless sensors (bandwidth of 20–450 kHz, 92 dB common mode rejection ratio and > 10^15^ Ω input impedance) transmitted the EMG signals to a desktop receiver (TeleMYO DTS EMG; Noraxon, Arizona, USA), and these were sampled at 2 kHz via an analogue-to-digital convertor (Micro 1401–3) and desktop PC utilizing Spike2 software (Cambridge Electronic Design, Cambridge, UK).

#### Transcranial magnetic stimulation (TMS) preparation

##### Optimal scalp position

The TMS preparation procedures followed relevant TMS methodological recommendations and were documented transparently based on a methodological reporting checklist^47^. Preparation for TMS data collection started by fitting the participant with a tight-fitting polyester cap that was used to mark the center of the scalp (Cz). With the vertex (Cz) positioned as the front right-most corner, a 3 × 3 cm grid was drawn on the left half of the head to identify the optimal scalp position (OSP) for stimulation of the right leg. A single-pulse double-cone coil TMS (110 mm external diameter) connected to Magstim 200^2^ magnetic stimulator (Magstim, Whitland, UK) was placed on the head. The coil was manually moved around the 3 × 3 cm grid in 0.5 cm intervals posterior and lateral from Cz in order to identify the OSP that produced the largest and most consistent MEPs in all three right-leg muscle groups (i.e., KE, PF, DF). The identified OSP was marked on the tight-fitted cap by tracing round the coil and this trace was referred to for consistent coil placement throughout the experiment.

##### Resting motor threshold

The resting motor threshold (RMT; excitability of the motor cortex at rest) of the individual was recorded by gradually reducing or increasing the stimulation intensity by 5% from the participants’ OSP intensity to find the minimum value capable of producing MEP amplitudes exceeding 50 μV in 5 of 10 consecutive trials^48^ in at least one of the muscles for each of the three muscle (i.e., KE, PF, DF). In accordance with previous research on AOMI^20,41^, the experimental stimulation intensity was set at 110% of the stimulation intensity used to identify RMT, with the intention to decrease direct wave stimulation^24,25^. The mean relative stimulator output intensity was 61 ± 11% for RMT and 68 ± 12% for the experimental protocol (i.e., 110% RMT stimulator output intensity).

#### Experimental setup

**Figure 6:**
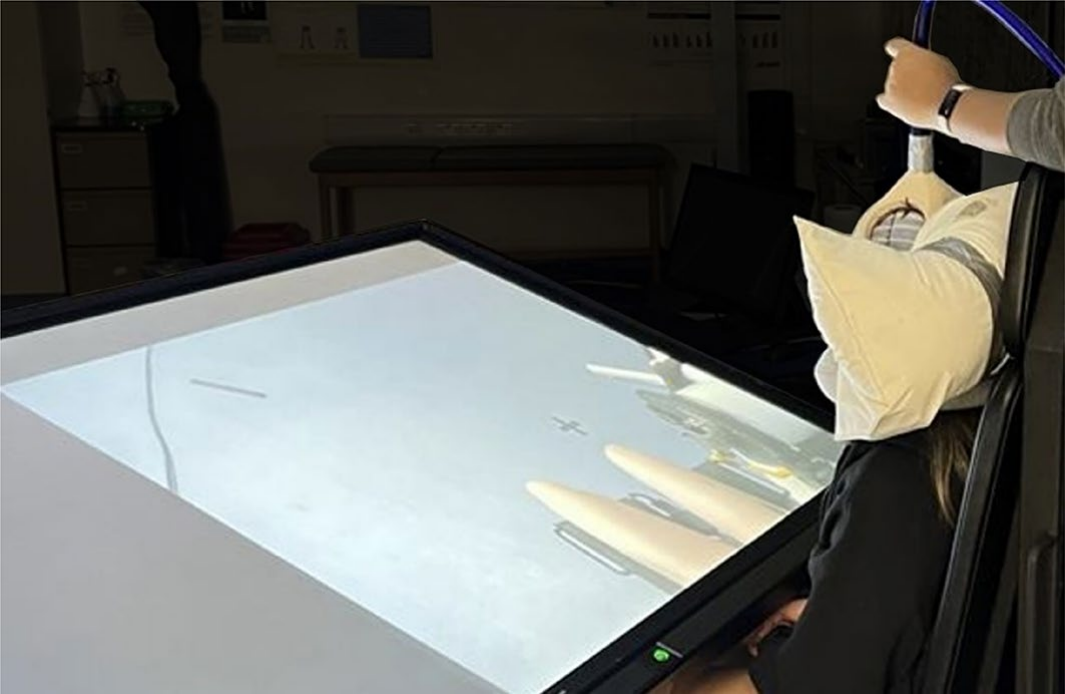
A Visual Representation of the Experimental Setup. *Note:* A visual representation of the experimental setup including the screen position, TMS coil placement and laboratory environment.

Following preparation for EMG recording and TMS delivery, participants began the experimental protocol (see Fig. 6). The participant was seated on a Norm isokinetic dynamometer chair (Cybex, New York, USA) in a dimly lit Biomechanics Laboratory at the host university, with their upper body loosely strapped to reduce trunk movement. The participant’s head was supported by a pillow to avoid movement and TMS coil repositioning during the experimental block, and maintain a consistent viewing position across experimental blocks. The participant’s feet were resting on stepping blocks adjusted to a comfortable height to ensure both legs were in a relaxed state, as monitored online for the right-leg by checking EMG activity was at baseline level. An 80″ adjustable LCD display was positioned over their lower body to ensure anatomical and perceptual congruency between the position of the participants’ legs and the observed legs on-screen in 1PP^49^. Mobile curtains were drawn alongside the experimental station to reduce the likelihood of visual distraction during data collection. Prior to beginning the experiment, participants were asked to read the on-screen instructions carefully, refrain from voluntary movement during the experimental blocks, and to attend fully to the stimuli presented and cognitive tasks implemented (see Fig. 5).

#### Experimental block preparation

Prior to each AOMI experimental condition, participants watched an instruction video to guide her/his engagement with the upcoming observed and imagined movements. The visual stimuli developed for use with this study were 1PP videos of a model performing isolated right-leg KE, PF, or DF movements. A female model was used for female participants, and a male model was used for male participants. All AOMI instruction videos were split-screen and displayed a 1PP video of a right-leg KE movement on the left side of the screen alongside a video of either a right-leg KE movement (AOMI_CONG_), right-foot PF movement (AOMI_COOR-FUNC_), or right-foot DF movement (AOMI_COOR-MOVE_) on the right side of the screen. The participants were provided with written and spoken instructions to observe the KE movement and simultaneously imagine the kinesthetic feelings and sensations involved with the right-leg KE, or right-foot PF or DF movement presented on the right side of the screen.

#### Experimental protocol

In a single testing session, participants completed five experimental blocks consecutively, with each block lasting around six minutes in total. A three-minute rest period was interspersed between blocks, and participants were encouraged to leave the testing chair to prevent eye strain and muscular discomfort before commencing with the next experimental block. The 30-trial experimental blocks were split into three sets of 10 trials, with written and verbal reminders of the block-specific instructions provided before the 1^st^, 11^th^ and 21^st^ trial per block. Every trial lasted 10 s and participants observed the stimuli shown on the LCD screen using DMASTR DMDX display software^50^. For the three AOMI conditions (see Fig. 5 for stimuli and instructions), the trials started by showing the model’s legs at rest (1000 ms), followed by four repetitions of the KE movement (2000 ms per cycle, 8000 ms per trial), before once again displaying the model’s leg at rest (1000 ms). When observing the KE movement, participants were instructed to simultaneously imagine the feelings and sensations associated with the imagined movement (i.e., either the KE, PF, or DF movement).

#### TMS data collection

Using a script run through Spike 2 software, a single TMS pulse was delivered once per trial at the point of maximum KE^51^. The TMS was delivered at the point of maximum KE for either the second (4000 ms) or third (6000 ms) cycle of the KE movement for all AOMI condition trials, and at the same time point for trials in both baseline conditions. The ordering of the TMS delivery was randomized and counterbalanced across trials for each experimental block. Two stimulation timings were used to reduce the predictability of the stimulation and subsequent anticipatory behavior of the participants^52^. A 3-s transition period was implemented between experimental trials to maintain an inter-stimulus interval greater than 10 s to let the effects of the preceding TMS pulse diminish^53^. Altogether, 30 stimulations were delivered to ensure a reliable measure of CSE per experimental condition^54–56^. Each experimental block was separated into three sets of 10 TMS trials by incorporating a standardized 15-s break between sets[e.g., ^20,28^]. This break period was implemented to maintain the participants’ attention levels and remind him/her of the condition-specific instructions for the experimental block. Participants’ visual attention was directed to the fixation cross and monitored online using an SMI Eye Tracking Glasses 2 Wireless system (SensoMotoric Instruments, Teltow, Germany). Participants visually attended to the fixation cross for all trials, meaning no trials were removed based on this screening step.

#### Social validation data collection

After the TMS data collection was finished, participants completed a social validation questionnaire and took part in a brief social validation interview developed for specific use in this study. Such bespoke social validation measures are commonly used in the applied sciences to understand participant perspectives regarding intervention effects^57^. More recently, TMS studies have adopted brief social validation questionnaires and interview schedules to understand potential cognitive and attentional mechanisms underlying engagement with AOMI (see e.g.,^20,58^). In this study, the participants outlined on three Likert scales from -2 (totally disagree) to 2 (totally agree) how much they felt like the leg that was presented to them throughout the experiment looked like their own leg, a male leg, and a female leg. Then, on three Likert scales ranging from 1 (very hard to feel) to 7 (very easy to feel), the participants rated the ease with which they were able to imagine the kinesthetic feelings and sensations for the imagined KE, PF and DF movements utilized in the different AOMI conditions. The semi-structured social validation interview checked for compliance with the intended manipulations and assessed the participants’ experiences of the experimental conditions. The interview guide consisted of four initial questions (e.g., “What imagery perspective did you employ across the experiment?”) and multiple probing questions (e.g., “Why? Did it change across conditions?”).

### Data analysis

#### TMS data processing

Peak-to-peak MEP amplitudes were recorded from the KE muscles (vastus lateralis and vastus medialis), PF muscles (soleus and gastrocnemius), and DF muscle (tibialis anterior) of the participants’ right leg on a trial-by-trial basis and averaged across all successful trials for each experimental condition. A two-part screening process was used to determine successful trials. First, trials were screened for the presence of an MEP response, and any trials where the MEP amplitude failed to reach 50 μV were removed. Second, to avoid MEP contamination by volitional muscle activity, EMG activity was recorded for 200 ms prior to each stimulation (see Table 3 for mean raw baseline EMG values recorded for each muscle group across the five experimental conditions) and any trials where the EMG amplitude exceeded normal baseline values for that experimental block (mean ± 2.5 SD) were also removed^20,28^. For the study sample, a mean value of 2.54 (± 3.49) trials for the KE muscle group, 1.88 (± 3.00) trials for the PF muscle group, and 1.75 (± 2.92) trials for the DF muscle group were removed per experimental condition. For each experimental condition, the number of successful trials per muscle group far exceeded the value needed (i.e., 24 trials) to provide a reliable estimate of CSE at rest^56^. On a muscle-by-muscle basis, the raw MEP amplitude data for successful trials (see Table 3 for mean raw MEP amplitude values recorded for each muscle group across the five experimental conditions) was normalized using a *z*-score transformation to account for the large intra- and inter-participant variability in MEP amplitudes at rest^20,23^. For each participant, this procedure involved standardizing the MEP amplitude value recorded for each successful trial against all other MEP amplitude values recorded for successful trials across the experimental protocol. The mean amplitude for all experimental trials was represented by a value of zero, and values for each experimental condition denoted by how many standard deviations that experimental condition was above or below the mean of all experimental conditions. Once the *z*-score transformation was complete, the muscle with the least removed trials across the experiment was selected to represent the KE muscle group (n = 10 vastus lateralis, n = 14 vastus medialis), PF muscle group (n = 14 soleus, n = 10 gastrocnemius), and DF muscle group (n = 24 tibialis anterior). In the case where both muscles from the same muscle group had an identical number of removed trials, the muscle with the lower peak-to-peak baseline EMG amplitude (μV) across the experiment was selected.

**Table 3:** Mean (± SD) raw baseline EMG and MEP amplitude values obtained for the knee extensor, plantar flexor, and dorsi flexor muscle groups in the human baseline (BL_H_), non-human baseline (BL_NH_), congruent AOMI (AOMI_CONG_), functionally-coupled coordinative AOMI (AOMI_COOR-FUNC_), and movement-direction coordinative AOMI (AOMI_COOR-MOVE_) experimental conditions.

EMG Measure	Muscle Group	Experimental Condition				
		Human Baseline	Non-Human Baseline	Congruent AOMI	Functionally-Coupled Coordinative AOMI	Movement- Direction Coordinative AOMI
Baseline EMG Amplitude (µV)	Knee Extensors	16.89 ± 2.94	16.71 ± 2.77	16.60 ± 2.67	16.56 ± 2.86	16.80 ± 2.84
	Plantar Flexor	16.27 ± 3.60	16.08 ± 3.31	16.79 ± 4.13	16.35 ± 3.63	16.32 ± 3.70
	Dorsi Flexor	17.69 ± 3.92	Table 317.72 ± 4.01	17.64 ± 4.27	17.95 ± 3.85	17.60 ± 3.82
MEP Amplitude (µV)	Knee Extensors	180.03 ± 112.07	176.11 ± 106.68	211.26 ± 145.81	199.20 ± 155.33	181.10 ± 120.57
	Plantar Flexor	195.65 ± 94.64	218.67 ± 120.34	257.20 ± 153.15	211.97 ± 99.05	195.16 ± 96.26
	Dorsi Flexor	623.41 ± 237.32	710.20 ± 215.75	625.03 ± 280.25	770.11 ± 261.99	616.24 ± 216.78

Raw MEP amplitude values reported above are of similar peak-to-peak amplitude to those reported in previous lower-limb TMS studies (e.g.,^59–61^).

#### TMS data analysis

The *z*-score MEP amplitude data for each muscle group was normally distributed, permitting the use of ANOVA statistical tests. Consequently, we analyzed the *z*-score MEP amplitude data for each muscle group using a separate one-way repeated measure analysis of variance (ANOVA) test with 5 levels (Experimental Condition: BL_H_, BL_NH_, AOMI_CONG_, AOMI_COOR-FUNC_, AOMI_COOR-MOVE_) in IBM SPSS Statistics 29 software package. Bonferroni contrasts were used for post-hoc pairwise comparisons. To test for potential contamination of z-score MEP amplitude data across experimental conditions, a separate one-way repeated measure analysis of variance (ANOVA) test with 5 levels (Experimental Condition: BL_H_, BL_NH_, AOMI_CONG_, AOMI_COOR-FUNC_, AOMI_COOR-MOVE_) was conducted on peak-to-peak baseline EMG values recorded across the 200 ms prior to TMS delivery. To test for a potential ordering effect across the experiment, a paired t-test was conducted for z-score MEP amplitude data recorded in experimental block 1 and experimental block 5, where participants completed 15 trials of both the BL_NH_ and BL_H_ experimental conditions. For this comparison, the z-score transformation was conducted based on the data from the 60 baseline trials collected for these two experimental blocks.

#### Social validation data analysis

A one-way repeated measures ANOVA with three levels (AOMI conditions: AOMI_CONG_, AOMI_COOR-FUNC_, AOMI_COOR-MOVE_) was used to examine participants’ ratings for ease of imagery across the different experimental conditions where imagery was instructed. The social validation interviews were transcribed and responses were grouped on a question-by-question basis to offer detailed explanations for the ease of imagery ratings from the social validation questionnaire.

## Data Availability

The datasets generated and analyzed during the current study are publicly available on the Open Science Framework: https://osf.io/6f2g8/.
